# Incorporating QM/MM Molecular Dynamics Into the Few‐Mode Quantization Approach for Light–Matter Interactions in Nanophotonic Structures

**DOI:** 10.1002/nap2.70099

**Published:** 2026-04-22

**Authors:** Ruth H. Tichauer, Maksim Lednev, Gerrit Groenhof, Johannes Feist

**Affiliations:** ^1^ Departamento de Física Teórica de la Materia Condensada Universidad Autónoma de Madrid Madrid Spain; ^2^ Condensed Matter Physics Center (IFIMAC) Universidad Autónoma de Madrid Madrid Spain; ^3^ Nanoscience Center and Department of Chemistry University of Jyväskylä Jyväskylä Finland

## Abstract

In the context of light–matter interactions between organic chromophores and confined photons of (plasmonic) nano‐resonators, we introduce a general framework that couples ab‐initio quantum mechanics/molecular mechanics (QM/MM) molecular dynamics with a recent quantization approach that allows to describe the electromagnetic field in nanophotonic environments with just a few modes. Arbitrary, lossy, and spatially inhomogeneous photonic environments are represented by a minimal set of interacting *photonic* modes fitted to the spectral density of the light‐confining structure, while geometry‐dependent molecular properties are computed on the fly. Applications to few‐molecule strong coupling show that strong light–matter interactions persist when molecular degrees of freedom and disorder are included for the chosen system consisting of a nanoparticle dimer coupled to five emitters. At the same time, symmetry‐protected degeneracies when modeling these five emitters as two‐level‐systems are lifted. The framework further reveals how spatial field inhomogeneity and molecular disorder shape cavity‐mediated energy transfer. This is illustrated for a donor–acceptor pair consisting in a photo‐reactive molecule that becomes resonant to the acceptor upon a light‐induced intramolecular proton transfer reaction.

## Graphical abstract

By incorporating an ab‐initio or first‐principles description of molecular emitters into a recent quantum optics approach that allows to accurately describe the electromagnetic field in nanophotonic environments, employing just a few quantised modes, we study the dynamics of realistic light–matter system with finite computational resources. Our approach further reveals how spatial field inhomogeneity and molecular disorder shape cavity‐mediated energy transfer.

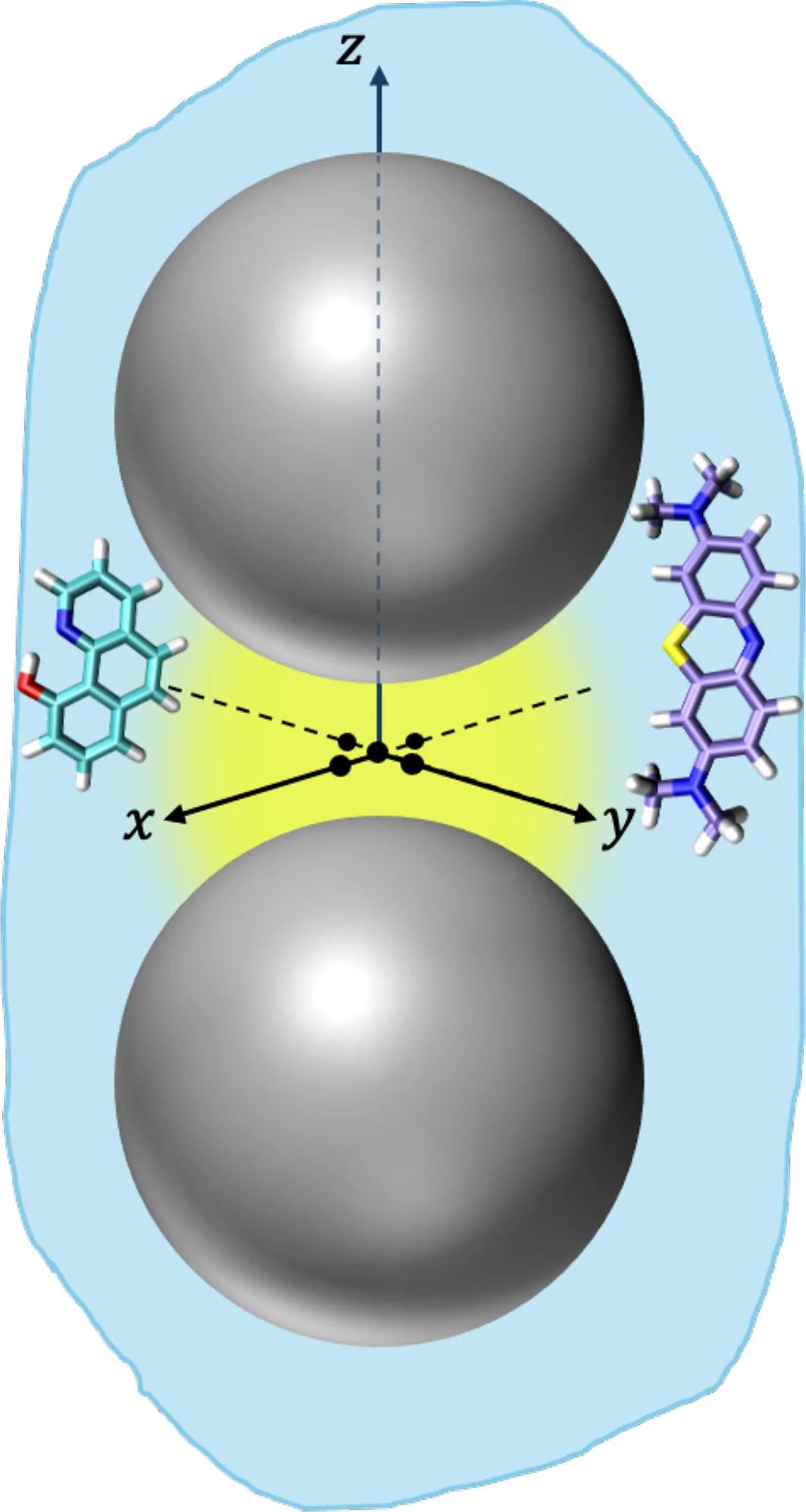

## 1 Introduction

Strong coupling between light and matter has emerged as a powerful framework to control molecular properties and chemical reactivity [1, 2]. When organic molecules are placed inside optical cavities and interact strongly with confined electromagnetic modes, hybrid light–matter states called polaritons form [3]. These polaritons exhibit unique properties that blend the characteristics of both photons and molecular excitations, opening new avenues for manipulating matter through vacuum fields [2]. Although strong coupling was initially demonstrated in Fabry–Pérot microcavities with large ensembles of molecules [4, 5], recent advances in plasmonic nanocavities have enabled strong coupling with only a few [6] or even single molecules [7] at room temperature.

Despite these experimental advances, theoretical descriptions of light–matter interactions at the nanoscale involving organic emitters face significant challenges due to the inherent complexity of such hybrid systems [8]. On the one hand, photoactive molecules possess many degrees of freedom, making their photo‐excitation dynamics dependent on both electrons and nuclei through nonadiabatic effects [9, 10, 11, 12]. The coupling of electronic and nuclear motion leads to the homogeneous broadening of electronic transitions, whereas interactions with the surrounding environment induce inhomogeneous broadening. On the other hand, electromagnetic fields confined at the nanoscale by nano‐resonators are highly inhomogeneous, with field amplitudes that depend on both frequency ω and position r [13, 14]. In plasmonic nanocavities, this spatial inhomogeneity is particularly pronounced, with field enhancements concentrated in nanometer‐scale “hot spots” [15, 16].

Several computational approaches have been developed in recent years to describe light–matter interactions involving photoactive molecules with high chemical accuracy [17, 18, 19, 20, 21, 22]. These methods range from studies of single molecules in gas phase coupled to single‐mode Fabry–Pérot resonators [18, 19, 20, 21] to treatments of molecules near plasmonic nanostructures [22]. However, most approaches either neglect environmental effects by considering isolated molecules [18, 19, 20, 21, 22] or approximate the electromagnetic environment as a single cavity mode [17, 18, 19, 20, 21].

To address the complexity associated to material systems, Luk et al. [17] pioneered a multiscale approach using quantum mechanics/molecular mechanics (QM/MM) [23] molecular dynamics (MD) [24] simulations to capture homogeneous and inhomogeneous broadening of molecular transitions as well as chemical reactivity. In this framework, the material part of the system is separated into QM and MM regions. Atoms in the quantum region are treated with ab‐initio electronic structure methods, meaning that the explicit electron cloud distribution is accounted for when determining the properties of this part of the system, such as the excitation energies and transition dipole moments. In contrast, the contribution of the surrounding environment is obtained accounting for the nuclear dynamics only. This is achieved by employing parametrized interaction potentials, usually referred to as classical force fields. The time evolution of the hybrid light–matter system follows a mixed quantum–classical dynamics scheme [9, 10, 11, 12], separating fast (electronic and photonic) and slow (nuclear) degrees of freedom following the Born–Oppenheimer‐like partitioning scheme leading to polaritonic potential energy surfaces [25, 26]. In this approach, the time‐dependent Schrödinger equation is solved for the coupled electron‐photon wavefunction Ψ(t) that parametrically depends on the nuclear coordinates R, whereas nuclear motion is governed by classical equations of motion in the potential landscape created by the fast (electron + photon) subsystem. Despite its sophistication in treating the molecular subsystem, this original implementation was limited to the collective coupling of many molecules to a single cavity mode.

We recently extended this QM/MM MD framework to multiple cavity modes [27], enabling the description of the full parabolic dispersion of Fabry–Pérot resonators. However, single‐molecule and few‐molecule strong coupling, which has been experimentally achieved in plasmonic nanocavities [6, 7], requires a treatment of the highly inhomogeneous and multimodal electromagnetic field distributions characteristic of such systems [15, 16].

In this work, we develop a general computational framework that combines QM/MM MD simulations with the few‐mode field quantization approach [28, 29] to accurately describe arbitrary nanophotonic structures. This method quantizes the continuous electromagnetic environment using a minimal set of interacting modes whose parameters are determined by fitting the spectral density obtained from classical electromagnetic simulations. We apply this framework to investigate how molecular degrees of freedom affect the excitation dynamics of a collection of photo‐active molecules in close proximity to plasmonic nanocavities. Our results show that strong light–matter coupling persists despite dynamic disorder introduced by nuclear motion and environmental fluctuations. However, symmetry‐based degeneracies that are preserved in idealized two‐level system (TLS) treatments of the emitters are lifted when realistic molecular properties are included. Furthermore, our approach enables the study of cavity‐mediated intermolecular energy transfer with full spatial resolution.

The paper is divided as follows: in Section 2, we present the model of light–matter interactions employing just a few modes for the confined electromagnetic field [28, 29]; in Section 3, we describe the methods and the systems employed in our simulations and, in Section 4, we present and discuss the obtained numerical results. We provide a summary and the implications of this work in the conclusions.

## 2 Theory

The Tavis–Cummings (TC) Hamiltonian [3] is a standard starting point to describe an ensemble of N identical two‐level emitters coupled to a single cavity mode within the rotating‐wave approximation (RWA):

(1) $${\hat{H}}^{TC}=\hbar \omega_{e}\sum_{i=1}^{N}{\hat{\sigma }}_{i}^{†}{\hat{\sigma }}_{i}+\hbar \omega_{c}{\hat{a}}^{†}\hat{a}-\hbar g\sum_{i=1}^{N}\left({\hat{a}}^{†}{\hat{\sigma }}_{i}+\hat{a}\;{\hat{\sigma }}_{i}^{†}\right),$$

with uniform emitter transition frequency $\omega_{e}$, single cavity frequency $\omega_{c}$, and identical light–matter coupling strength g for all emitters.

For organic chromophores placed near nanophotonic structures, almost all the assumptions inherent to the TC model are typically violated: (i) Molecular emitters are not identical two‐level systems, since nuclear motion and heterogeneous environments induce time‐dependent, molecule‐specific excitation energies and transition dipoles. (ii) The electromagnetic environment is strongly multimodal and lossy, making a single‐mode description or even a description based on a few independent modes insufficient. (iii) For each photonic mode, the field is highly inhomogeneous, so couplings differ from emitter to emitter.

Instead of undoing the approximations that went into the TC model step by step, we therefore start directly from a much more general description based on few‐mode quantization for arbitrary nanophotonic structures [28, 29, 30], which does not rely on any assumptions on the structure or nature of the photonic modes or the emitters, but instead is based on the theory of macroscopic QED [31] to describe arbitrary electromagnetic environments. The method relies on the observation that the cavity‐mediated dynamics and interaction between emitters is fully determined by the spectral density of the electromagnetic environment. This in turn is determined by the dyadic Green's function $G\left(r_{i},r_{j},\omega \right)$, specifically its imaginary part for reciprocal media, as we will assume here (for nonreciprocal media, it generalizes to the anti‐Hermitian part [32, 33]):

(2) $$J_{ij}\left(\omega \right)=\frac{\omega^{2}}{\hbar \pi \epsilon_{0}c^{2}}\;\text{Im}\;\left[G\left(r_{i},r_{j},\omega \right)\right].$$

Here $r_{i}$ and $r_{j}$ are the positions of emitters i and j, respectively, $\epsilon_{0}$ is the vacuum permittivity, and c is the speed of light in vacuum. $J_{ij}\left(\omega \right)$ is a matrix in Cartesian components of the electric field vector for each combination of emitter indices i, j. Note that we here chose a slightly different presentation of the method than in previous works, which allows the description of arbitrary dipole orientations more naturally. We then construct a minimal, interacting few‐mode model that reproduces $J_{ij}\left(\omega \right)$ as closely as possible, described by the Hamiltonian:

(3) $${\hat{H}}_{F}=\sum_{i=1}^{N}{\hat{H}}_{i}^{e}+\sum_{k,l}\hbar \omega_{kl}{\hat{a}}_{k}^{†}{\hat{a}}_{l}-\sum_{i,k}{\hat{\mu }}_{i}\cdot g_{ik}\left({\hat{a}}_{k}+{\hat{a}}_{k}^{†}\right),$$

where ${\hat{H}}_{i}^{e}$ is the Hamiltonian of emitter i, ${\hat{\mu }}_{i}$ its transition dipole operator, ${\hat{a}}_{k}^{†}$ $\left({\hat{a}}_{k}\right)$ the creation (annihilation) operator of the quantized photonic mode k, $\omega_{kl}$ the mode‐mode coupling matrix, and $g_{ik}$ the electric field strength of mode k at the position of emitter i. The Hamiltonian ${\hat{H}}_{i}^{e}$ can be arbitrarily defined. Later on, we will adopt a QM/MM definition of this Hamiltonian. The photonic modes are lossy and the system dynamics are then described by a Lindblad master equation,

(4) $$\dot{\rho }=-i\left[H_{F},\rho \right]+\sum_{k}\kappa_{k}L_{a_{k}}\left[\rho \right],$$

where ρ is the system density matrix, $L_{O}\left[\rho \right]=O\rho O^{†}-\frac{1}{2}\left\{O^{†}O,\rho \right\}$ is a Lindblad dissipator, and $\kappa_{k}$ is the decay rate of mode k. The spectral density of this few‐mode model, $H_{F}$, is given by [29]

(5) $$J_{ij}^{\text{mod}}\left(\omega \right)=\frac{1}{\pi }\sum_{kl}g_{ik}\text{Im}\;{\left[\frac{1}{\tilde{H}-\omega }\right]}_{kl}g_{jl},$$

where ${\tilde{H}}_{kl}=\omega_{kl}-\frac{i}{2}\kappa_{k}\delta_{kl}$. The real parameters $\omega_{kl}$, $\kappa_{k}$, and $g_{ik}$ of the few‐mode quantization model, ${\hat{H}}_{F}$, are then obtained by the nonlinear fitting of the effective spectral density, $J_{ij}^{\text{mod}}\left(\omega \right)$, to the full one $J_{ij}\left(\omega \right)$ in Equation (2), which is calculated by solving Maxwell's equations. This procedure yields a compact photonic Hamiltonian without assuming spatial homogeneity, a single mode, or identical emitters. Although the diagonal terms in $\tilde{H}$ could be regarded as Lorentzian photonic modes, we emphasize that the total $J_{ij}^{\text{mod}}\left(\omega \right)$ is not a sum of Lorenzians and can only faithfully reproduce $J_{ij}\left(\omega \right)$ by including the interaction terms $\omega_{kl}$ of $\tilde{H}$.

Up to this point, the nature of the emitter has not been specified—it is simply described by its Hamiltonian ${\hat{H}}_{i}^{e}$ and dipole operator ${\hat{\mu }}_{i}$. In most previous works on few‐mode quantization [13, 28, 29, 30, 34, 35], the emitters were modeled as two‐level systems. Here, we go beyond this simplified description and model the molecular subsystem using the approach of QM/MM molecular dynamics for polaritonic systems [17, 27]. In this framework, each molecule i is treated as a few‐level system (restricted to two levels in the applications below), whose properties depend on the nuclear coordinates $R_{i}$ of that molecule at each time step of the MD simulation. We thus compute on the fly, that is to say at each time‐step, the geometry‐dependent ground‐ and excited‐state energies $V_{S_{0}}\left(R_{i}\right)$ and $V_{S_{1}}\left(R_{i}\right)$, as well as the transition dipole $\mu_{i}\left(R_{i}\right)$ between them. Applying the RWA to the general light–matter Hamiltonian then leads to our working model for the light–matter dynamics:

(6) $$\begin{array}{c} {\hat{H}}^{LM}=\sum_{i=1}^{N}\left[V_{S_{0}}\left(R_{i}\right)+\hbar \omega_{i}^{e}\left(R_{i}\right){\hat{\sigma }}_{i}^{†}{\hat{\sigma }}_{i}\right]+\sum_{kl}\hbar \omega_{kl}{\hat{a}}_{k}^{†}{\hat{a}}_{l}-\sum_{i,k}\mu_{i}\left(R_{i}\right)\cdot g_{ik}\left({\hat{\sigma }}_{i}^{-}{\hat{a}}_{k}^{†}+{\hat{\sigma }}_{i}^{+}{\hat{a}}_{k}\right), \end{array}$$

where $\hbar \omega_{i}^{e}\left(R_{i}\right)=V_{S_{1}}\left(R_{i}\right)-V_{S_{0}}\left(R_{i}\right)$ is the excitation energy of molecule i at geometry $R_{i}$, and ${\hat{\sigma }}_{i}^{+}$ $\left({\hat{\sigma }}_{i}^{-}\right)$ is the raising (lowering) operator between the electronic ground and excited state of molecule i. In contrast to the TC model, this Hamiltonian retains the multimode structure of the EM field, spatially varying couplings, and the full molecular complexity captured by QM/MM MD.

We perform simulations starting with a single excitation in the system. Since the Hamiltonian conserves the number of excitations, and the only terms that do not conserve the number of excitations in the Lindblad master equation are the so‐called quantum jump or refilling terms $\propto a_{k}\rho a_{k}^{†}$ (which make population that decays from the single‐excitation subspace “reappear” in the ground state manifold), the dynamics within the single‐excitation subspace is equivalently determined by the non‐Hermitian Schrödinger equation with Hamiltonian ${\tilde{\hat{H}}}^{LM}={\hat{H}}^{LM}-\frac{i}{2}\sum_{k}\kappa_{k}{\hat{a}}_{k}^{†}{\hat{a}}_{k}$. We thus work with a wavefunction‐based approach within the single‐excitation subspace. We expand the total electron–photon wavefunction as $|\Psi \left(t\right)\rangle =\sum_{m=1}^{N+n}c_{m}\left(t\right)|\psi^{m}\rangle$, where $|\psi^{m}\rangle$ is the state with a single excitation in component m of the system while all others are in their ground state (m=1,…,N being the molecules, and m=N+1,…,N+n being the photonic modes). $c_{m}\left(t\right)$ is the time‐dependent amplitude of each state $|\psi^{m}\rangle$.

In contrast to the fast electrons and photons, the dynamics of the slow nuclei are determined by integrating Newton's equations of motion. We incorporate the influence of the electromagnetic field on nuclear motion by including in the MD force evaluation contributions arising from light–matter couplings to the confined modes. Within the mean‐field Ehrenfest framework [9, 10, 36], each atom a in molecule j experiences a Hellmann–Feynman force gradient that supplements the electronic (QM) or force‐field (MM) potential:

(7) $$F_{HF\to a\in j}^{MF}=-|c_{j}\left(t\right){|}^{2}\nabla_{a}V_{S_{1}}^{j}\left(R_{j}\right)-\sum_{m\neq j}^{N+n}|c_{m}\left(t\right){|}^{2}\nabla_{a}V_{S_{0}}^{j}\left(R_{j}\right)+\sum_{k=0}^{n-1}\left(c_{j}^{*}\left(t\right)c_{k}\left(t\right)\nabla_{a}\mu \left(R_{j}\right)\cdot g_{jk}+c.c.\right).$$

Equation (7) applies when working in a diabatic basis $\left\{|\psi^{m}\rangle \right\}$ of the hybrid light–matter system, constructed as the tensor product of uncoupled molecular states $|S_{0}^{0}\text{…}S_{1}^{i}\text{…}S_{0}^{N}\rangle$ and photonic Fock states $|0_{0}\text{…}1_{k}\text{…}0_{n-1}\rangle$ [27, 37]. This diabatic basis is equivalent to the unique adiabatic basis consisting of the polaritonic eigenstates of the full light–matter Hamiltonian in Equation (6). In Section I of the Supporting Information S1, we provide the corresponding Hellmann–Feynman force expressions in the adiabatic representation. Both formulations are implemented in our simulation code.

## 3 Methods

### 3.1 Organic Chromophores

We investigate three distinct photoactive molecules: rhodamine (Rho), methylene blue (MeB), and 10‐hydroxybenzo[h]quinoline (HBQ), whose structures are shown in the insets of Figures 1a and 5a. Prior to the few‐mode QM/MM MD simulations described in Section 4, all molecular systems underwent a two‐stage preparation protocol: initial optimization, solvation, and equilibration at the classical molecular mechanics (MM) level, followed by refinement at the QM/MM level.

**FIGURE 1:**
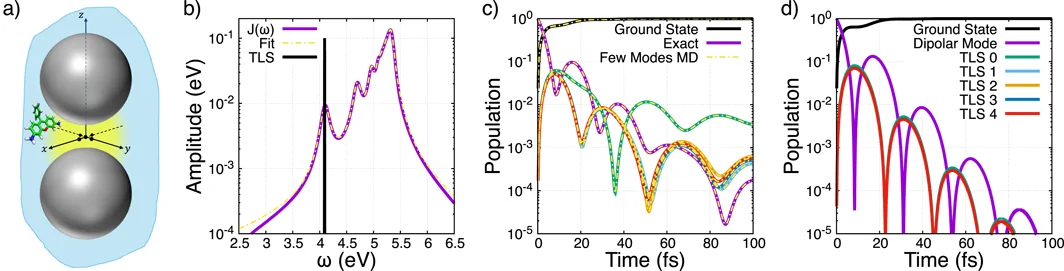
(a) Schematic representation of five point‐like emitters within the gap of a silver nanoparticle dimer oriented along the z−axis. When accounting for the molecular degrees of freedom, a rhodamine model depicted in the inset is placed at each spot. (b) Physical (plain purple line) and fitted (yellow pointed‐dashed line) spectral densities of the central emitter with its dipole moment oriented along the z‐axis. The absorption of the emitters corresponds to a delta function (black vertical line) when treated as ideal two‐level systems (TLSs). (c) Time‐evolution of the population of the five TLSs resonantly coupled to the dipolar mode of the optical resonator when the electromagnetic environment is described with 40 modes. (d) Same population dynamics but when the electromagnetic environment within the nanocavity is described by a single mode corresponding to the dipolar mode.

For the classical MM simulations, we employed the Amber03 force field [38] combined with the TIP3P water model [39] for rhodamine, using parameters developed by Luk et al. [17]. Methylene blue utilized parameters from Sokolovskii et al. [40], whereas HBQ solvated in cyclohexane was described with the GROMOS‐2016H66 force field [41]. Detailed protocols for the MM‐level optimization, solvation, and equilibration of rhodamine can be found in Tichauer et al. [27] and those for methylene blue and HBQ in Sokolovskii et al. [40].

Following MM preparation, all systems were equilibrated at the QM/MM level using an MD time step of 1 fs. Consistent with previous implementations [17, 27], the QM region of rhodamine comprised the three fused rings, treated at the restricted Hartree–Fock (RHF) level with the 3‐21G basis set. The remainder of the molecule (benzene side group) and the aqueous solvent constituted the MM region, described by the Amber03 force field [38] and TIP3P water model [39], respectively. For methylene blue and HBQ, the entire molecular framework was treated quantum‐mechanically. Ground‐state electronic properties were computed using density functional theory (DFT) [42] with the ωB97X‐D [43] and CAM‐B3LYP [44, 45] functionals for methylene blue and HBQ, respectively, both employing the 6‐31G* basis set [46]. Methylene blue was solvated in water (TIP3P model [39]), whereas HBQ was solvated in cyclohexane (GROMOS‐2016H66 force field [41]). To account for environmental electrostatic effects, the QM regions of rhodamine and methylene blue were electrostatically embedded in their respective MM environments, experiencing the Coulomb field of all MM atoms within the cutoff radii of 1.6 and 1.0 nm, respectively. Lennard‐Jones interactions between QM and MM atoms provided mechanical coupling for all systems. Temperature control was achieved using the velocity‐rescaling thermostat [47] with a coupling time constant of 0.1 ps, applied every 100 MD steps.

After QM/MM system preparation, excited‐state potential energy surfaces $V_{S_{1}}^{QM/MM}\left(R\right)$ were constructed on‐the‐fly during the few‐mode QM/MM Ehrenfest MD simulations by computing the first singlet excited state $\left(S_{1}\right)$ at each time step. The QM/MM partitioning schemes established for ground‐state calculations were maintained for excited‐state treatments. For rhodamine, the QM region was described using configuration interaction singles (CIS) with the 3‐21G basis set. We note, however, that this level of theory leads to a large over‐estimation of its excitation energy. This does not affect our results since we employ this rhodamine model for investigating the effects of dynamic disorder rather than for investigating the photo‐physics of the chromophore itself. For methylene blue and HBQ, we employed time‐dependent DFT (TD‐DFT) [48] with the ωB97X‐D and CAM‐B3LYP functionals, respectively, both with the 6‐31G* basis set. The MM environments were modeled using the Amber03 force field [38] with the TIP3P water model [39] for rhodamine, TIP3P water model alone for methylene blue, and the GROMOS96‐2016H66 force field [41] for the cyclohexane solvent surrounding HBQ.

All molecular simulations, both in vacuum and within the nano‐resonator, were performed using GROMACS 4.5.3 [49] interfaced with TeraChem [50] for the QM/MM treatment of the material subsystem within the hybrid light–matter system.

### 3.2 Photonic Resonator

The photonic resonator employed in this work consists of a silver nanosphere dimer oriented along the z‐axis. Each sphere has a 20‐nm radius such that the two particles are separated by a 1.5‐nm gap (see Figures 1a and 5a).

Using the Drude model for the frequency‐dependent relative permittivity ϵ(r,ω) of silver, with literature values for the different parameters [51, 52], and a permeability μ(r,ω)=1, we computed the electromagnetic dyadic Green's function $G\left(r_{i},r_{j},\omega \right)$ for this nanostructure using the SCUFF‐EM boundary element method code [53, 54]. The boundaries of each nanosphere constituting the dimer were provided by a mesh generated by the finite element mesh generator gmesh [55]. In our model geometry, five emitters are positioned within the dimer gap: one at the center and four 1 nm away at symmetrically equivalent peripheral locations forming a square centered on the first emitter (Figures 1a and 5a). All emitters lie in the equatorial plane of the gap, with a minimum distance of 0.75nm from each nanoparticle surface to prevent quantum tunneling that would arise from the wavefunction overlap between the molecular emitters and the metallic nanoparticles.

Following the numerical computation of $G\left(r_{i},r_{j},\omega \right)$, we fitted the resulting spectral density $J_{ij}\left(\omega \right)$ (Equation 2) using a 40‐mode representation. The fitting procedure was iterated until achieving a convergence criterion of $10^{-4}$ in the relative error. In the electromagnetic simulations, the dipole mode of the nanoresonator appears as a peak at 2.3 eV, resonant with the absorption of actual Rhodamine molecules. However, at the level of theory we use for Rhodamine, this absorption is blue‐shifted to 4.1 eV. We compensate for this over‐estimation by shifting the entire spectral density by 1.8 eV. Such a constant shift does not induce any artifacts as we employ the RWA and restrict the excitation dynamics to the single excitation subspace, such that all dynamics only depend on energy differences within that subspace.

Both the exact and fitted spectral density are depicted in Figure 1b, with the dipole moment of the central emitter oriented along the z‐axis.

## 4 Results and Discussion

### 4.1 Lindblad Master Equation Versus Few‐Mode QM/MM MD for TLSs

To validate the few‐mode QM/MM MD implementation, we start by modeling the five emitters in the setup described in Subsection 3.2 as identical ideal TLSs and compare the dynamics of the excited state populations of this hybrid light–matter system to the dynamics obtained, employing the well‐established Lindblad master equation of open quantum systems [56]. These five TLSs are set to have an excitation energy of 4.10 eV and transition dipole moment equal to 9.42 D, comparable to our rhodamine molecular model. They are resonantly coupled to the bright dipolar mode [52] of the silver nanosphere dimer (see Figure 1b). The confined electromagnetic field within this nano‐resonator is modeled with 40 modes such that the first one corresponds to the dipolar mode. This well separated peak can be fitted with a single Lorentzian at $\hbar {\tilde{\omega }}_{0}=4.10$ eV with a width of $\hbar \kappa_{0}=160$ meV that corresponds to a decay time of $1/\kappa_{0}=8.23$ fs. We fix the parameters of $J^{\text{mod}}\left(\omega \right)$ (Equation 5) such that this fitted dipolar mode is decoupled from the rest of the modes, that is, the off‐diagonal terms in the first row/column of $\tilde{H}$ are set equal to zero (${\tilde{\omega }}_{0i}={\tilde{\omega }}_{i0}=0$ for i∈[1,n−1], where n=40 is the number of photonic modes).

Agreement with error below $10^{-4}$ is observed between the few‐modes QM/MM MD implementation and the Lindblad master equation in the population dynamics of the five TLSs coupled to the 40‐mode silver nanoparticle dimer (see Figure 1c and Supporting Information S1: Figure S1). Although the initial excitation of the bright dipolar mode (purple line in Figure 1c) inevitably decays over time, concomitant with a monotonous increase of the ground state occupation (black line) that reflects the total losses in the system, the population of this dipolar mode oscillates coherently with that of the five TLSs, evidencing strong light–matter coupling [57]. In other words, as the population of the dipolar mode decreases, that of the TLSs increases, and vice versa. The population of the central emitter in Figure 1c (green line) differs from that of the four peripheral emitters (light blue, orange, dark blue, and red lines), which remain identical within a TLS description. Their equivalence can be understood by their symmetrically equivalent positions with respect to the central emitter and the silver nanoparticle dimer. Small differences arise from the nonsymmetric meshing of the nanoparticle dimer used to numerically compute the dyadic Green's function $G\left(r_{i},r_{j},\omega \right)$. If desired, symmetry can be enforced in the electromagnetic environment, but this is not done here for the sake of generality of the approach.

We additionally computed the excitation dynamics when modeling the confined electromagnetic field with a single mode (Figure 1d). This single mode corresponds to the dipolar mode to which the five emitters are resonantly coupled and has the same parameters as the decoupled fitted dipolar mode. The population dynamics in Figure 1d show that a single‐mode approximation is far from accurate for this system. Even though in the 40‐mode fit $J^{\text{mod}}\left(\omega \right)$, the dipolar mode $\left({\tilde{\omega }}_{0},\kappa_{0}\right)$ is decoupled from the other 39 modes and dominates radiation to the far field [52], a single‐mode model cannot reproduce the clear difference in dynamics between the central and peripheral emitters observed in Figure 1c. The remaining 39 fitting modes $\left(\left\{{\tilde{\omega }}_{k},\kappa_{k}\right\}\right)$, together with their mutual couplings $\left\{{\tilde{\omega }}_{kp}\right\}$, are responsible for representing the highly inhomogeneous electromagnetic field within the dimer gap that is essential to capture the correct dynamics. In a single‐mode description, Figure 1d, the difference between emitters is reduced to a different coupling strength to that mode, which cannot capture the full complexity of the system. We note, however, that an accurate single‐cavity mode approach has been recently developed but requires weak enough couplings between the emitter and nonresonant cavity modes. These couplings can then be treated within a Markovian approximation that retains the qualitative physics while enabling an effective single‐mode treatment [35]. We do not explore this possibility further here, as our focus is on the full few‐mode description. Moreover, since for organic chromophores the excitation energies fluctuate due to nuclear motion and environmental effects, a distinction between resonant and nonresonant modes is not straightforward.

### 4.2 Effect of Incorporating Molecular Degrees of Freedom

Having validated the few‐mode QM/MM MD approach for TLSs and quantified the accuracy of our implementation against the Lindblad master equation (see Figure 1c and Supporting Information S1: Figure S1), we next examine how molecular degrees of freedom (homogeneous broadening) and solvent interactions (inhomogeneous broadening) affect light–matter interactions.

To assess the impact of internal molecular degrees of freedom, we first employ our rhodamine model in gas phase (full QM simulations, no solvent) at several temperatures (0 K, 4 K, 10 K, 20 K, and 100 K). As described in Subsection 3.1 , the ground $\left(S_{0}\right)$ and first excited $\left(S_{1}\right)$ states of the QM region (fused rings of rhodamine) were treated at the RHF/3‐21G and CIS/3‐21G levels of theory, respectively. Although the five emitters share the same initial conformation (i.e., no initial diagonal or off‐diagonal disorder as in the TLS case), including internal nuclear motion results in markedly different population dynamics compared to a TLS treatment (Figures 1c and 2).

**FIGURE 2:**
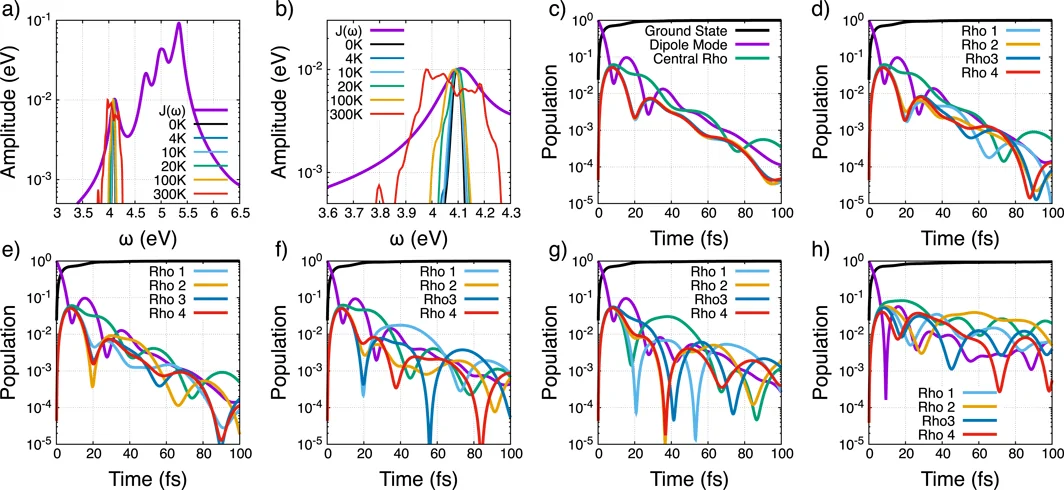
(a) Fitted spectral densities of the hybrid light–matter system shown alongside the absorption of the molecular emitters in gas phase at various temperatures and in solution at 300 K. (b) Zoom into the dipolar mode of the spectral density and its overlap with the molecular absorption under different conditions. (c) Time evolution of the populations of the rhodamines, the dipolar mode of the silver nanoparticle dimer, and the overall ground state (system losses) for gas‐phase emitters at 0 K. (d) Same populations at 4 K, (e) 10 K, (f) 20 K, (g) 100 K, and (h) in solution (water, QM/MM model) at 300 K.

At 0 K, no vibrational modes are populated initially. Yet, differences emerge between the TLS treatment and the full‐QM gas‐phase simulations (Figures 1c and 2c). These differences stem from the finite kinetic energy acquired during MD. During the few‐mode QM MD simulation, the initial energy deposited in the resonator dipolar mode is partially converted into vibrational energy through light–matter interactions with the confined field. Because the field enhancement is maximal at the center of the gap, the central rhodamine attains an average kinetic energy corresponding to an effective temperature of ∼1.5 K, whereas the peripheral molecules heat to ∼0.25 K (see Supporting Information S1: Figure S5). This temperature difference reflects the unequal electromagnetic field intensities experienced within the nanocavity. By adjusting the coupling time constant of the velocity‐rescaling thermostat, atomic velocities can be rescaled so that the average kinetic energy matches the target temperature.

Increasing the temperature in gas‐phase simulations (4 K, 10 K, 20 K, and 100 K; Figure 2d–g) progressively lifts the degeneracy among the four peripheral emitters, whose populations cease to oscillate coherently earlier in time (∼35, 30, 15, and 12 fs for 4 K, 10 K, 20 K, and 100 K, respectively). This effect originates from dynamic disorder. Different initial atomic velocities, randomly sampled from a Maxwell–Boltzmann distribution, drive distinct explorations of the vast phase space of rhodamine (37 atoms, yielding 105 vibrational modes). At 0 and 4 K (Figure 2c,d), the effective light–matter coupling is reduced relative to the TLS case, as evidenced by less pronounced population oscillations, whereas at 10 K, 20 K, and 100 K, the coupling is enhanced (panels e–g). From 10 K onward, the increased homogeneous broadening due to higher vibrational occupation improves the spectral overlap between the molecular excited‐state resonance and the resonator dipolar mode (Figure 2a,b), thereby strengthening the effect of the light–matter coupling such that it overcomes the additional disorder introduced by nuclear motion. Strong coupling is maintained across all temperatures considered.

Including inhomogeneous broadening by modeling the five emitters in solution (explicit water using the TIP3P model at 300 K) further lifts the degeneracy among the four peripheral molecules, whose excited‐state populations remain superimposed only during the first ∼7 fs (Figure 2h). Strong coupling persists despite the substantial disorder introduced by the solvent. However, at 300 K in solution, the interaction strength is reduced compared to gas‐phase simulations at 10 K, 20 K, and 100 K. The molecular absorption (red curve in Figure 2b) broadens sufficiently that the ensemble explores regions of phase space no longer resonant with the resonator dipolar mode.

As mentioned above, because of the vast phase space available to molecular emitters, we conducted 100 additional few‐mode QM/MM MD simulations of solvated rhodamine within the dimer gap. As shown in Figure 3, coherent population dynamics are maintained in individual realizations but, when averaged, persist only for ∼20–30 fs, with two pronounced oscillations for the molecules (panels a–e) and three for the bright dipolar mode (panel f). In individual realizations, the degeneracy among the four peripheral emitters is lifted by distinct nuclear trajectories, but on average, the four peripheral positions are equivalent. Nevertheless, the dynamic disorder introduced by solution‐phase MD is insufficient to make the central and peripheral dynamics equivalent. Thus, despite the small spatial extent of the ensemble (the four peripheral emitters are only 1 nm from the central one), they experience, on average, different electromagnetic environments within the nanoparticle dimer gap. A single‐mode approximation would miss this key feature, which is critical for the design of molecule‐nanocavity devices.

**FIGURE 3:**
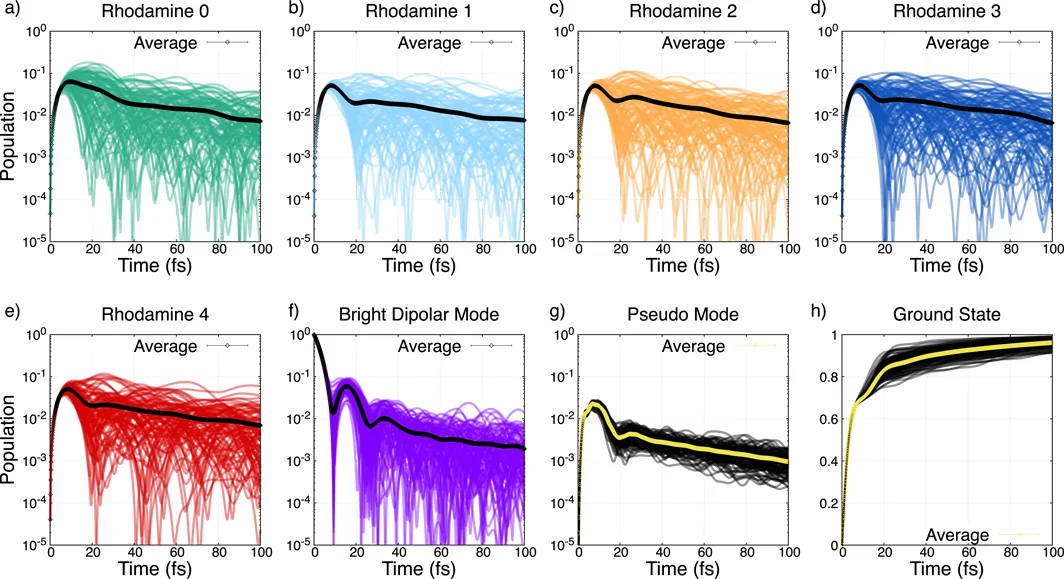
(a) Time evolution of the excited‐state population of the central rhodamine molecule within the setup of Figure 1a across 101 few‐mode QM/MM MD simulations. The average over these 101 realizations is shown with black diamonds. (b–e) Time evolution of the populations of the four peripheral molecular emitters. (f) Population dynamics of the dipolar mode of the silver nanoparticle dimer. (g) Population dynamics of the modes forming the broad pseudo‐mode (peak at ∼5.35 eV in Figure 1b). The average over the 101 realizations is shown with yellow diamonds. (h) Ground‐state occupation reflecting losses of the silver nanoparticle dimer. Note the linear scale in this panel.

### 4.3 Energy Transfer Mediated by a Nanocavity

Organic semiconductor materials are increasingly integrated into electronic technologies, despite inherent disorder that limits efficiency [58]. For the performance of organic solar cells, LEDs, and transistors, energy transfer between chromophores is often crucial. In this context, Gonzalez‐Ballestero et al. [59] predicted that exciton transfer between two‐level emitters can be enhanced under strong coupling to nanocavity hot spots. Leveraging our approach that goes beyond a TLS description of the emitters, we thus also investigate cavity‐mediated intermolecular energy transfer.

In the setup of Figure 1a, rather than initially exciting the bright dipolar mode that couples to the far field [52], we consider an initial excitation localized on the central rhodamine chromophore. The populations of the molecular emitters, the resonator dipolar mode, and the overall ground state across 100 few‐mode QM/MM MD simulations are shown in Figure 4.

**FIGURE 4:**
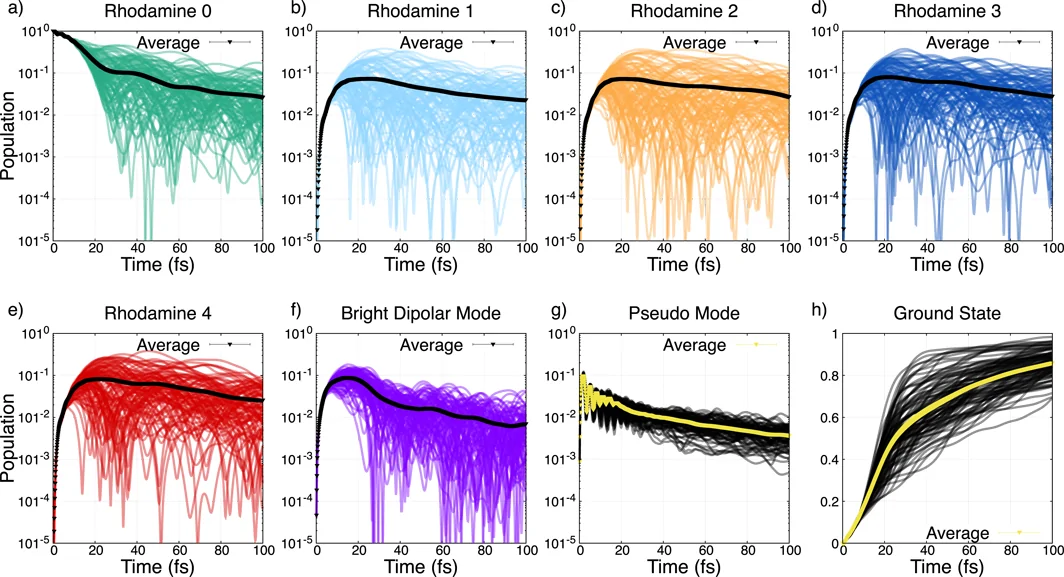
(a) Time evolution of the excited‐state population of the central rhodamine in the setup of Figure 1a for 100 few‐mode QM/MM MD simulations with initial excitation on the central molecule. The average over these 100 realizations is shown with black diamonds. (b–e) Time evolution of the populations of the four peripheral molecular emitters. (f) Population dynamics of the dipolar mode of the silver nanoparticle dimer. (g) Population dynamics of the modes forming the broad pseudo‐mode (peak at ∼5.35 eV in Figure 1b). The average over the 100 realizations is shown with yellow diamonds. (h) Ground‐state occupation due to losses of the silver nanoparticle dimer. Note the linear scale in this panel.

**FIGURE 5:**
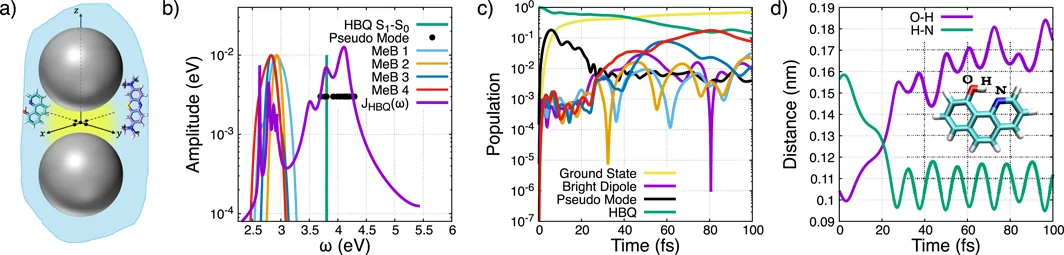
(a) Schematic of five molecular emitters within the gap of a silver nanoparticle dimer oriented along the z‐axis. HBQ (left inset) occupies the central site, and methylene blue molecules (right inset) occupy the peripheral positions. (b) Effective spectral density perceived by HBQ shown with the absorption of methylene blue (light blue, orange, dark blue, and red lines). Black dots indicate the fitted‐mode frequencies ${\tilde{\omega }}_{k}$ forming the pseudo‐mode in our model. (c) Time evolution of the excited‐state populations of the five emitters (HBQ: green; MeB1: light blue; MeB2: orange; MeB3: dark blue; MeB4: red), together with that of the bright dipolar mode (purple) and the pseudo‐mode (black; broad peak at ∼4 eV). The ground‐state population (yellow) increases steadily due to system losses. (d) O–H and H–N distances in HBQ (inset). An H–N distance of ∼0.105 nm corresponds to a bonded state.

Relative to a TLS treatment (Supporting Information S1: Figure S6), energy transfer from the central rhodamine to the peripheral ones is significantly enhanced when molecular degrees of freedom are included. In the TLS case, peripheral emitters reach at most ∼4%–5% occupation by 15 fs and decay to ∼2% by the end of the simulation, whereas with nuclear motion and solvent interactions (Figure 4), individual realizations reach up to ∼40% (∼10% on average) at ∼30–40 fs before decaying to ∼10% (∼3%–4% on average) by 100 fs. This enhancement arises from increased spectral overlap between rhodamine absorption and the resonator dipolar mode once homogeneous and inhomogeneous broadening are included.

Since the fluorescence lifetime of rhodamine is on the order of nanoseconds, radiative molecular decay to free‐space background modes is negligible on the timescale of our simulations (hundreds of femtoseconds). Consequently, both intermolecular energy transfer and overall excitation decay are mediated mostly through the nanocavity modes, and in particular the dipolar mode, $\left({\tilde{\omega }}_{0},\kappa_{0}\right)$. The average loss rate is therefore reduced when initially exciting the central rhodamine instead of the dipolar mode: on average, it takes ∼80 fs for the ground state population to reach 80% (Figure 4h), compared to ∼15 fs when initially exciting the dipolar mode (Figure 3h). We note, however, that initially exciting the central rhodamine (Figure 4) leads to an initial ∼10% occupation of the so‐called pseudo‐mode (Figure 4g), that is, the broad peak at ∼5.35 eV that is due to the combined action of high‐order modes of the nanoparticles [52], compared to ∼2%–3% in (Figure 3g). Thus, while total losses are reduced, nonradiative channels mediated by the pseudo‐mode play a more prominent role for localized initial excitations.

### 4.4 Photochemistry‐Driven Energy Transfer

To mimic experimental conditions with a substantial disorder at room temperature (300 K), the rhodamine chromophores in the simulations shown in Figure 4 were assigned different initial conformations and velocities, and thus different excitation energies. Experimentally, however, selectively exciting the central rhodamine (or a specific peripheral emitter) would be challenging because, on average, the emitters share the same absorption spectrum (Supporting Information S1: Figure S6b). We therefore replace the central rhodamine by HBQ (left inset, Figure 5a) and the peripheral emitters by methylene blue (right inset, Figure 5a). We also shift the fitted spectral density $J^{\text{mod}}\left(\omega \right)$ associated with the silver nanoparticle dimer so that our methylene blue model is resonant with the dipolar mode $\left({\tilde{\omega }}_{0},\kappa_{0}\right)$, here set to ${\tilde{\omega }}_{0}=2.89$ eV (Supporting Information S1: Figure S9). Such shift enables a straightforward comparison with the energy transfer scheme studied with rhodamine chromophores but now also including a photochemistry drive.

Upon far‐blue/UV absorption, HBQ undergoes an ultrafast excited‐state intramolecular proton transfer reaction [60, 61]. The associated conformational change (H bonded to N instead of O; see inset in Figure 5d) produces a large Stokes shift with emission peaking in the green–yellow region. Because the absorption maximum of methylene blue is in the same spectral region (Supporting Information S1: Figure S7), this donor–acceptor pair is well suited to study photochemistry‐driven, cavity‐mediated intermolecular energy transfer, similar to the recent proposal of photochemistry‐driven polariton propagation by Sokolovskii et al. [40] using the same molecular pair. Critically, our approach enables photochemical reactions in cavities owing to the QM/MM treatment of the material subsystem. Although prior implementations were restricted to 2D Fabry–Pérot microresonators [27], the present extension accommodates photonic resonators of arbitrary shape.

As shown in Supporting Information S1: Figure S8, a TLS treatment precludes energy transfer due to minimal spectral overlap between the donor and acceptor (HBQ absorption at ∼3.8 eV, MeB at ∼2.8 eV in our model). Although within a TLS approximation, it would be possible to set the energy of the central emitter “manually” to that of the post‐reaction HBQ state (i.e., resonant with the peripheral TLSs and/or the dipolar mode), this would overlook the substantial role played by the pseudo‐mode in this configuration, as shown next.

We performed simulations with an initial excitation localized on solvated HBQ (cyclohexane, 300 K) at the center of the nanoparticle dimer gap (Figure 5a). To provide additional context, in Figure 5b, we show the *effective* spectral density $J_{\text{HBQ}}\left(\omega \right)$ perceived by HBQ when treating the four MeB molecules as part of the “photonic” environment felt by the HBQ molecule within a linear response. Here, the four solvated methylene blue (MeB) molecules (water, 300 K) had distinct initial structures (static diagonal/off‐diagonal disorder) and velocities (dynamic disorder), sampled from a Maxwell–Boltzmann distribution at 300 K. Following blue/UV excitation of HBQ, its excited‐state population (green) decreases monotonically (Figure 5c). Concomitantly, the pseudo‐mode population (black), which aggregates higher‐order cavity modes [52] and several fitting modes in our few‐mode approach (black dots in Figure 5b), increases, as does the ground‐state occupation that reflects total losses. Around ∼20 fs, the pseudo‐mode population drops sharply, whereas the bright dipolar mode (purple) rises by nearly an order of magnitude within ∼5 fs. This transition coincides with the completion of HBQ's intramolecular proton transfer (Figure 5d), after which the HBQ excited state experiences a Stokes shift into resonance with the dipolar mode. Because MeB absorption overlaps with this dipolar mode (Supporting Information S1: Figure S9), the MeB ensemble becomes strongly coupled to it. The MeB populations (light blue, orange, dark blue, and red) then begin coherent oscillations with the dipolar mode and rise by nearly an order of magnitude within another ∼5 fs. By 100 fs, MeB populations reach ∼13% of the total excitation, with MeB4 (red) reaching up to ∼8% (Figure 5c).

When considering an idealized scenario in which the MeB emitters share identical initial structures at their absorption maximum (Supporting Information S1: Figure S10), the cavity‐mediated energy transfer is not necessarily enhanced relative to the disordered case of Figure 5. This underscores the decisive role of dynamic disorder in ensembles of molecular emitters.

To further explore the phase space for the HBQ + 4 MeB system, we performed additional simulations with different initial velocities sampled from a Maxwell–Boltzmann distribution at 300 K. Although these results (Supporting Information S1: Figure S11) corroborate the mechanism of cavity‐mediated energy transfer between reactive HBQ and MeB, the total population accruing on the peripheral emitters remains small relative to overall losses (ground state reaching ∼70%; yellow curve in Figure 5c and Supporting Information S1: Figure S11h). Future work will optimize this photochemistry‐driven, cavity‐mediated energy transfer setup in silico.

## 5 Conclusions

Bridging recent advances in quantum optics [28, 29] and quantum chemistry [27] into a unified numerical framework opens new opportunities for in silico control of light–matter interactions at the nanoscale. Leveraging high‐performance computing and few‐mode quantization of the electromagnetic field [28, 29], our implementation brings quantum optics and quantum chemistry together without sacrificing accuracy for multichromophoric systems coupled to confined fields in plasmonic nanostructures. Using this tool, we show that strong coupling persists in the presence of substantial static and dynamic disorder introduced by nuclear motion and that molecular degrees of freedom lift degeneracies preserved in TLS descriptions. We further demonstrate cavity‐mediated intermolecular energy transfer and highlight the role of pseudomodes in loss channels. These advances pave the way for the in silico design of molecule‐nanocavity architectures and the integration of organic emitters into photonic circuits [62].

## Author Contributions

R.H.T. and J.F. conceptualised the work with contributions from G.G. R.H.T. and M.L. performed the simulations and analysed the data under the supervision of J.F. R.H.T. developed the QM/MM MD code and wrote the initial manuscript. Computational resources were provided and managed by J.F. All authors reviewed and approved the final version of the manuscript.

## Supporting information


Supporting Information S1

## Data Availability

The data that support the findings of this study are openly available in Zenodo at https://doi.org/10.5281/zenodo.18300234, reference number 18300234.
