# Supplementary material for: Incorporating QM/MM Molecular Dynamics Into the Few‐Mode Quantization Approach for Light–Matter Interactions in Nanophotonic Structures
Source: Nanophotonics. 2026 Apr 22;15(8):e70099. doi: 10.1002/nap2.70099 (PMC13642168; doi:10.1002/nap2.70099)
Supplement: Supplementary file 1 [file NAP2-15-e70099-s001.pdf]

Supporting Information for "Incorporating QM/MM molecular dynamics into the few-mode quantization approach for light-matter interactions in nanophotonic structures"

Ruth H. Tichauer*[1,2], Maksim Lednev[1,2], Gerrit Groenhof[3], and Johannes Feist†[1,2]

[1]Departamento de Física Teórica de la Materia Condensada, Universidad Autónoma de Madrid, E-28049 Madrid, Spain

[2]Condensed Matter Physics Center (IFIMAC), Universidad Autónoma de Madrid, E-28049 Madrid, Spain

[3]Nanoscience Center and Department of Chemistry, University of Jyväskylä, P.O. Box 35, Jyväskylä, 40014, Finland

Contents

I Quantum Mechanics/ Molecular Mechanics Molecular Dynamics **2**

II Ehrenfest force in the adiabatic basis **2**

III Validation of the few-mode QM/MM MD implementation **2**

III.1 Comparison with the Lindblad master equation . . . 2

III.2 Energy conservation . . . 3

IV Additional results **5**

IV.1 Effect of incorporating molecular degrees of freedom . . . 5

IV.2 Initially exciting the central emitter . . . 5

IV.3 Photochemistry-driven energy transfer . . . 6

*ruth.tichauer@uam.es

†johannes.feist@uam.es

I Quantum Mechanics/ Molecular Mechanics Molecular Dynamics

Molecular Dynamics (MD) [1], is a quantum chemistry approach that allows to study the time-dependent behavior of an ensemble of interacting elementary units (atoms/molecules/unit cell) by integrating Newton's equations of motion. Commonly, the interacting potentials between the arbitrarily chosen units, are parametrized to reproduce macroscopic experimental properties of the material under study and are referred to Molecular Mechanics (MM) force fields.

While MM MD has proven useful in the atomistic study of systems counting tens of thousands of atoms such as crystalline bulk materials, macro-molecules, nanoparticles or cell-membranes, the developed force fields are fixed meaning that in-situ electronic re-arrangements that would lead to bond formation and breaking, involved in chemical reactions, cannot occur.

To add such capacity while still allowing the simulation of large realistic systems, in 1976, a Quantum Mechanics/Molecular Mechanics (QM/MM) partitioning was proposed [2]. In this QM/MM scheme, the interaction potential between atoms belonging to the QM region is determined quantum mechanically, hence accounting explicitly for electrons and their wave-like nature, while the interaction potential within the MM region is still retrieved from the parametrized force fields. Various schemes exist for gluing together the QM and MM regions. As such, QM/MM integrates quantum effects only where needed, offering both accuracy and efficiency in the simulation of large, realistic systems.

II Ehrenfest force in the adiabatic basis

In the theory section of the main text (section II), we presented the Hellmann-Feynman force for mean-field Ehrenfest dynamics [3] in the diabatic basis of uncoupled molecular and photonic excitations. We here additionally show the same forces when working in the *adiabatic* basis of the light-matter Hamiltonian consisting of its (polaritonic) eigenstates, $|\Psi(t)\rangle = \sum_{p=1}^{N+n} c_p(t)|\phi_p\rangle$, with $|\phi_p\rangle = \sum_{m=1}^{N} \beta_m^p|\psi_m\rangle + \sum_{m=N+1}^{N+n} \alpha_k^p|\psi_m\rangle$. The forces are then given by

$$\begin{aligned}
\mathbf{F}_{HF\to a\in m}^{\text{adiabatic}} &= \left[|c_p(t)|^2|\beta_m^p|^2 + \left(c_p^*(t)c_q(t)\beta_m^{p*}\beta_m^q + c.c.\right)\right]\nabla_{a\in m}V_{S_1}(\mathbf{R}_m)\\
&+\left[|c_p(t)|^2\left(\sum_{j\neq m}|\beta_j^p|^2 + \sum_k|\alpha_k^p|^2\right) - \left(c_p^*(t)c_q(t)\beta_m^{p*}\beta_m^q + c.c.\right)\right]\nabla_{a\in m}V_{S_0}(\mathbf{R}_m)\\
&-|c_p(t)|^2\left(\beta_m^{p*}\sum_k\alpha_k^p + c.c.\right)\nabla\mu(\mathbf{R}_m)\cdot\mathbf{g}_{jk}\\
&-c_p^*(t)c_q(t)\sum_k\left(\beta_m^{p*}\alpha_k^q + \beta_m^q\alpha_k^{p*}\right)\nabla\mu(\mathbf{R}_m)\cdot\mathbf{g}_{jk}\\
&-c_p(t)c_q^*(t)\sum_k\left(\beta_m^p\alpha_k^{q*} + \beta_m^{q*}\alpha_k^p\right)\nabla\mu(\mathbf{R}_m)\cdot\mathbf{g}_{jk}.
\end{aligned} \tag{S1}$$

III Validation of the few-mode QM/MM MD implementation

III.1 Comparison with the Lindblad master equation

To validate the present implementation that incorporates QM/MM MD into the few-mode approach [4, 5], we start by modeling molecular emitters as ideal TLSs within the setup depicted in Fig. S1a. By integrating the time-dependent Schrödinger equation, in either the diabatic or locally diabatic basis [6] (the latter is employed when working in the adiabatic basis of H^{LM}, Eq. (6)), we compute the time evolution of the total (polaritonic) wavefunction of the hybrid light-matter system

$$|\Psi(t)\rangle = \sum_{p=1}^{N+n} c_p(t)|\psi^p\rangle \tag{S2}$$

In the above expression, $c_p(t)$ are the time-dependent expansion coefficients and ψ^p are the basis states.

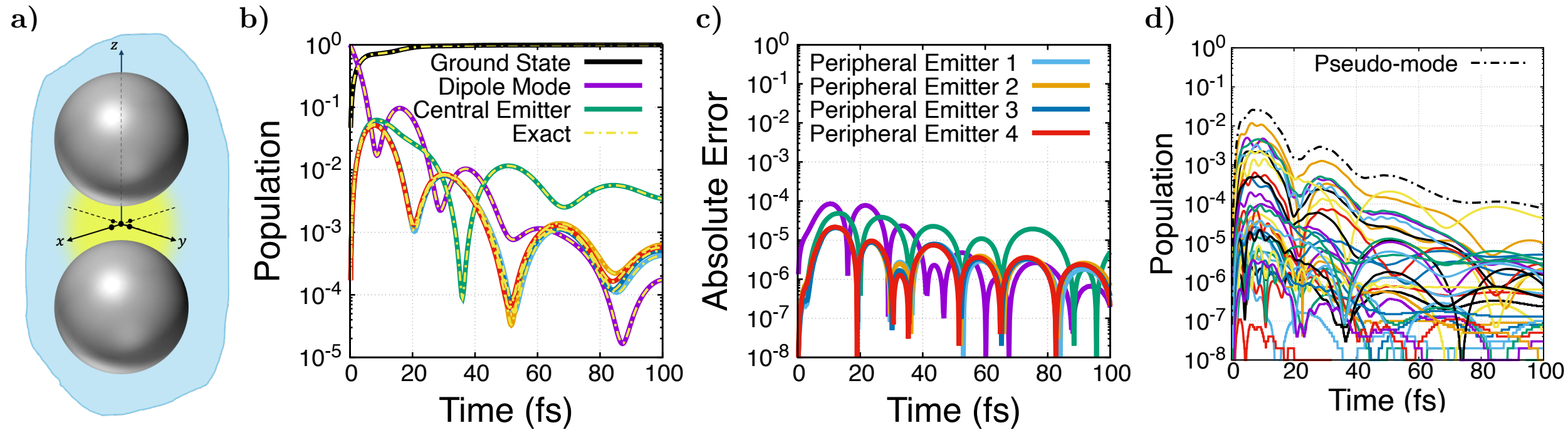

Figure S1: (a) Schematic representation of five point-like emitters, modeled as ideal two-level systems, within the gap of a silver nanoparticle dimer oriented along the z-axis. (b) Time evolution of the populations of the five emitters resonantly coupled to the dipolar mode of the optical resonator. (c) Absolute error between the population dynamics obtained with our integrator and with the Lindblad master equation, labeled as the "exact" reference method. (d) Time evolution of the population of modes 1 to 39 of the nano-resonator.

We then compute the populations $\rho_p(t) = c_p^*(t)c_p(t)$ of the diabatic or adiabatic states ψ^p. Comparison between the population dynamics obtained with the present implementation and the well-established Lindblad master equation of open quantum systems for TLSs [7] shows that the absolute difference between these two integrators is on the order of 10^{-4}-10^{-5}, as depicted in Fig. S1b and Fig. S1c.

While in Fig. S1b the population of the dipolar mode of the nanoresonator is depicted in purple, the population of the other photonic modes are represented in Fig. S1d. We have summed the populations of modes constituting the so-called pseudo-mode [8] and plotted with a dashed-pointed black line.

III.2 Energy conservation

As previously done and for comparison with the former implementation beyond a single-mode approximation [9], we test the numerical implementation of the forces by performing full-QM gas-phase Ehrenfest simulations (time step $dt = 0.1$ fs) of one, two, and four Rhodamine chromophores (S_0 and S_1 states described at the HF/3-21G and CIS/3-21G levels of theory, respectively) coupled to lossless 2D Fabry-Pérot microcavities described by 1, 10, 20, and 100 modes ($\omega_0 = 3.95$ eV, $L = 5$ μm length, field amplitude $E_0 = 0.002$ au). In Fig. S2 and Fig. S3 we show that the total energy is conserved, i.e., no energy drift as a function of time. Importantly, the energy fluctuations are independent of the number of confined photon modes, i.e., same amplitude for cavities described by 1, 10, 20, and 100 cavity modes, as depicted in Fig. S2.

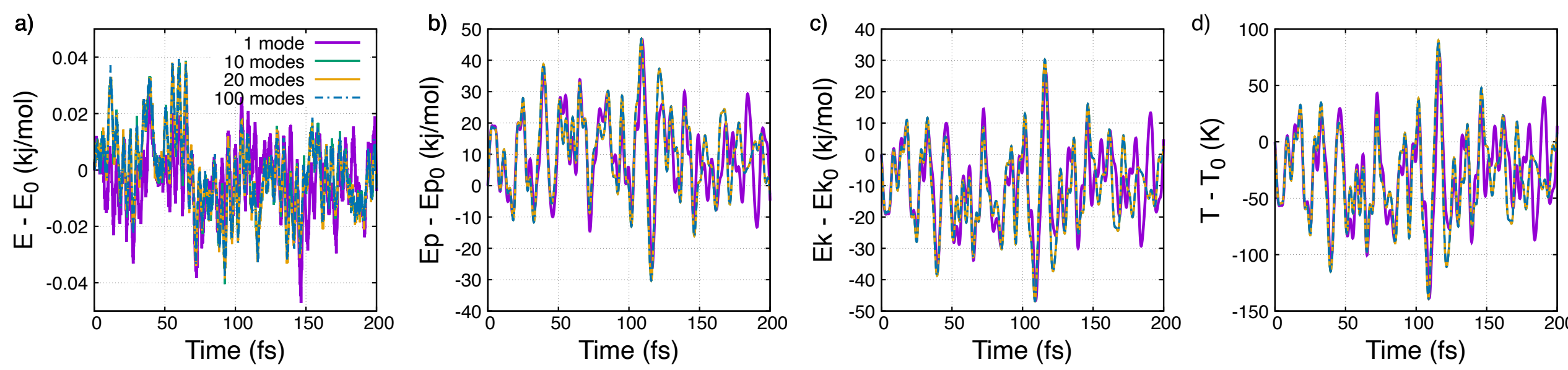

Figure S2: Fluctuations in the (a) total, (b) potential, and (c) kinetic energy, as well as in the (d) temperature, during few-mode (full-QM) MD simulations of one Rhodamine chromophore in gas phase coupled to lossless 2D Fabry-Pérot microcavities described by 1 (purple), 10 (green), 20 (orange), and 100 (blue dashed) cavity modes. These simulations were run in the diabatic basis of uncoupled molecular and photonic excitations ($|S_0^0 \dots S_1^i \dots S_0^N\rangle \otimes |0_0 \dots 1_k \dots 0_n\rangle$).

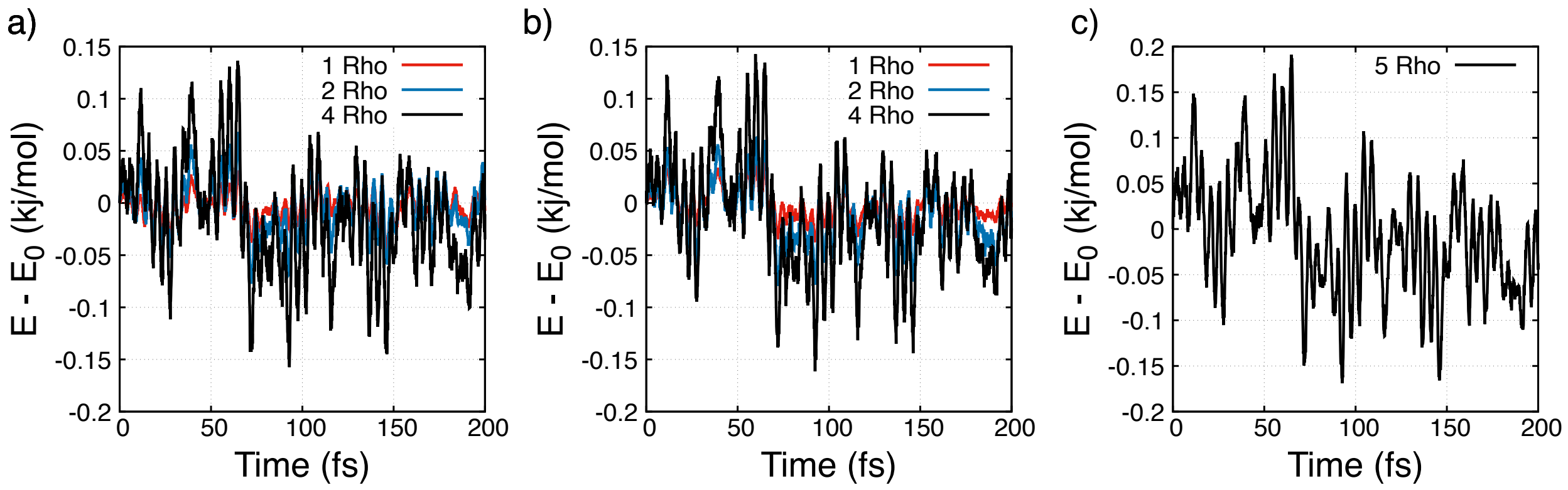

Figure S3: Fluctuations in the total energy during few-mode (full-QM) MD simulations of one (red), two (blue), and four (black) Rhodamine chromophores in gas phase coupled to lossless 2D Fabry-Pérot microcavities described by (a) a single cavity mode and (b) 10 cavity modes. The simulations in (a) were run in the diabatic basis of uncoupled molecular and photonic excitations ($|S_0^0 \dots S_1^i \dots S_0^N\rangle \otimes |0_0 \dots 1_k \dots 0_n\rangle$) while the simulations in (b) were run in the adiabatic basis of the light-matter Hamiltonian, Eq. (6). (c) Fluctuations of the total energy of the system depicted in Fig. S4a when omitting losses of the nanoresonator.

Running simulations in the adiabatic basis of the light-matter Hamiltonian, Eq. (6), is more computationally demanding than running the same simulation in the diabatic basis of uncoupled molecular and photonic excitations ($|S_0^0 \dots S_1^i \dots S_0^N\rangle \otimes |0_0 \dots 1_k \dots 0_n\rangle$ [9, 10]) because of the step involved in diagonalizing the matrix. We ensure that energy is conserved irrespective of the chosen basis. In Fig. S3b we present energy conservation tests for simulations in the adiabatic basis.

We also performed gas-phase (full QM) MD simulations at 0 K of five Rhodamine chromophores coupled to the field confined by a silver nanoparticle dimer. As depicted in Fig. S4, the absolute difference in the excited-state populations are on the order of 10^{-8}-10^{-7} when running in the diabatic and adiabatic basis of the light-matter Hamiltonian (Eq. (6)). When omitting the losses of the nanoresonator, the total energy is conserved (Fig. S3c)

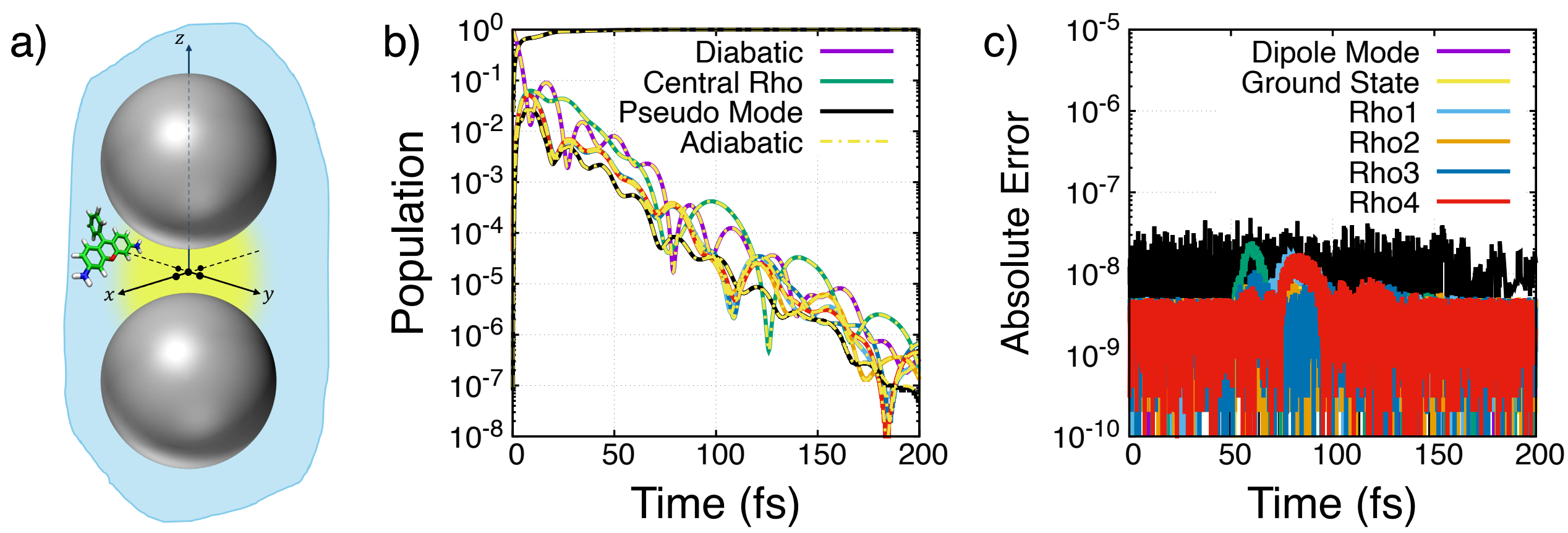

Figure S4: (a) Schematic representation of five Rhodamine chromophores within the gap of a silver nanoparticle dimer oriented along the z-axis. (b) Excited-state populations of the central (green) Rhodamine, the peripheral (light blue, orange, blue, and red) molecular emitters, and the dipolar mode of the nanoresonator (purple) computed from simulations in the diabatic basis of uncoupled molecular and photonic excitations ($|S_0^0 \dots S_1^i \dots S_0^N\rangle \otimes |0_0 \dots 1_k \dots 0_n\rangle$). These same populations obtained from simulations in the adiabatic basis of the light-matter Hamiltonian, Eq. (6), are plotted with yellow dashed lines. (c) Absolute difference in the populations when running simulations in the diabatic and adiabatic basis.

IV Additional results

IV.1 Effect of incorporating molecular degrees of freedom

We performed numerical simulations of the hybrid light-matter system depicted in Fig. 1a of the main article with identical initial conditions (excitation energies, transition dipole moments) but treating the five Rhodamine molecules as *(i)* ideal two-level systems (Fig. 1 in the main), *(ii)* in the gas phase (full QM) at different temperatures (Fig. 2 in the main), and *(iii)* in solution (QM/MM) at 300 K (Fig. 3 in the main).

For controlling temperature during MD simulations, we employed the velocity rescaling algorithm [11] with a time constant coupling of 0.1 ps applied every 100 steps of the simulation (time step $dt = 0.1$ fs). During the few-mode (full QM) gas-phase simulation at 0 K, the temperature of the different emitters is slightly higher than that set by the MD thermostat but stabilizes around a fixed value: approximately 0.25 K for the peripheral and approximately 1.5 K for the central Rhodamine as depicted in Fig. S5.

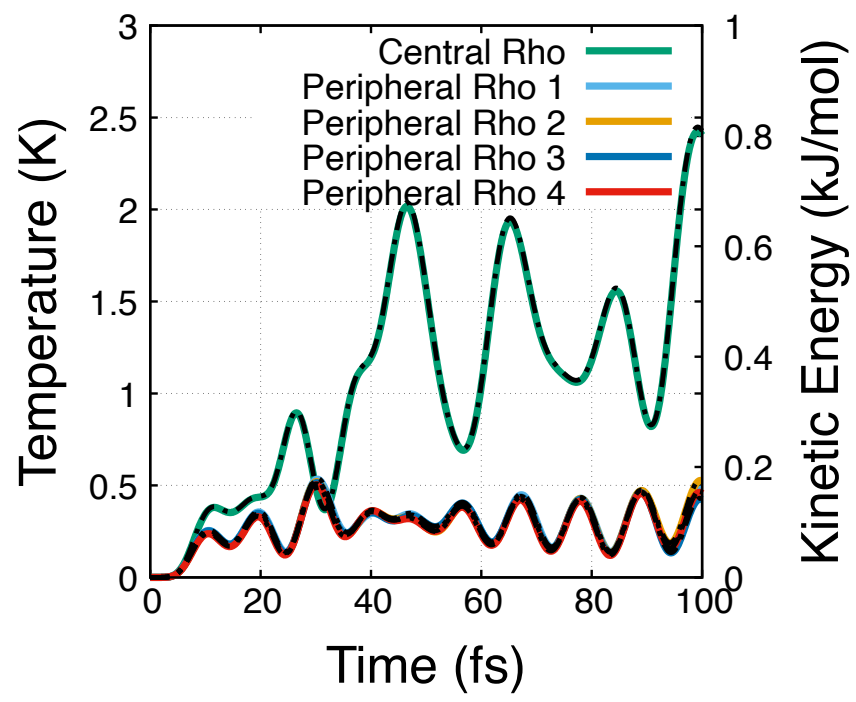

Figure S5: Temperature during gas-phase few-mode (full QM) MD simulations at 0 K. The kinetic energy, in kJ/mol, as a function of time is also plotted with black dashed-dotted lines.

IV.2 Initially exciting the central emitter

Following Gonzalez-Ballestero et al. [12], we investigate cavity-mediated intermolecular energy transfer within a two-level system approximation (Fig. S6c) and including the molecular degrees of freedom (Fig. 4 in the main article and Fig. S6d).

While the simulation presented in Fig. S6(d) does not correspond to the best case scenario where dynamic disorder enhances intermolecular energy transfer maximally, the QM/MM MD treatment of the emitters leads to higher occupation of one of the peripheral emitters (approximately 10% at 20 fs) than the two-level system treatment (approximately 4% at 16-17 fs). This is due to the broadening of the molecular resonance that thus has higher overlap with the dipole mode of the nanoresonator, resulting in stronger light-matter coupling.

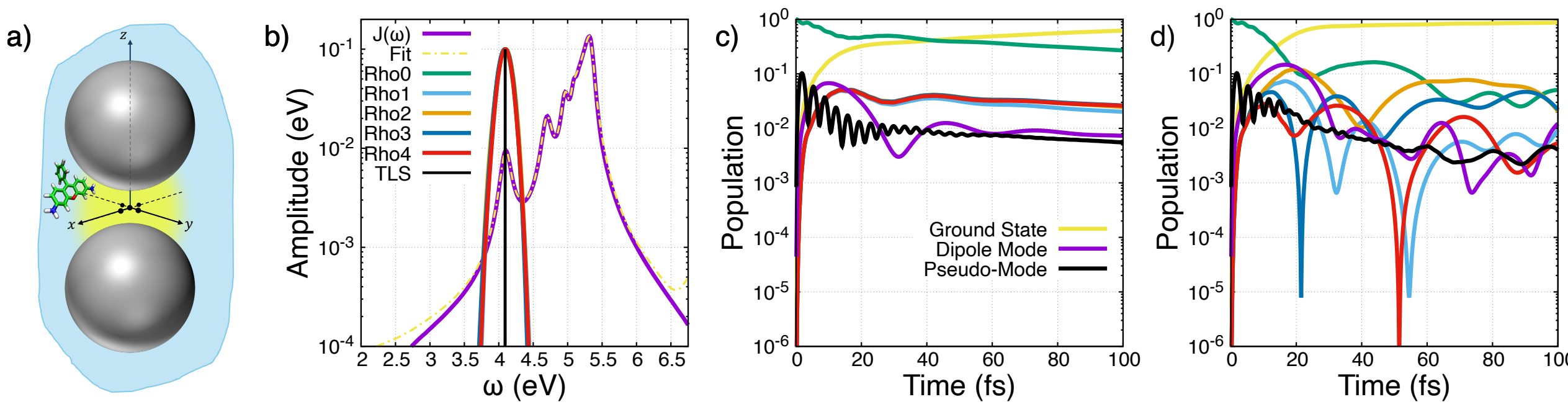

Figure S6: (a) Schematic representation of five point-like emitters within the gap of a silver nanoparticle dimer oriented along the z-axis. (b) Physical (purple) and fitted (yellow dashed) spectral densities of the central emitter with its dipole moment oriented along the z-axis plotted together with the absorption spectra of the Rhodamine model (green, light blue, orange, dark blue, and red). When treated as ideal two-level systems (TLS), the absorption of these molecular emitters corresponds to a delta function (black vertical line). (c) Time evolution of the populations of the five emitters, modeled as ideal TLS, resonantly coupled to the dipolar mode of the optical resonator when initially exciting the central emitter. (d) Same populations but accounting for the molecular degrees of freedom during a few-mode QM/MM MD simulation with identical initial parameters as the TLS treatment.

IV.3 Photochemistry-driven energy transfer

As shown in Fig. S7, the overlap between the QM/MM absorption spectrum of Methylene Blue (ωB97X-D/6-31G*/TIP3P TD-DFT level of theory) and the photoluminescence (PL) spectrum of HBQ (CAM-B3LYP/6-31G*/Gromos-2016H66 TD-DFT level of theory) makes these two molecular emitters suitable models for investigating, *in silico*, photochemistry-driven donor-acceptor intermolecular energy transfer mediated by cavity modes.

We note that the photoluminescence spectrum of HBQ strongly depends on when the intramolecular proton transfer reaction takes place. The sooner this chemical reaction completes, the higher the left peak and the smaller the right peak in its PL spectrum.

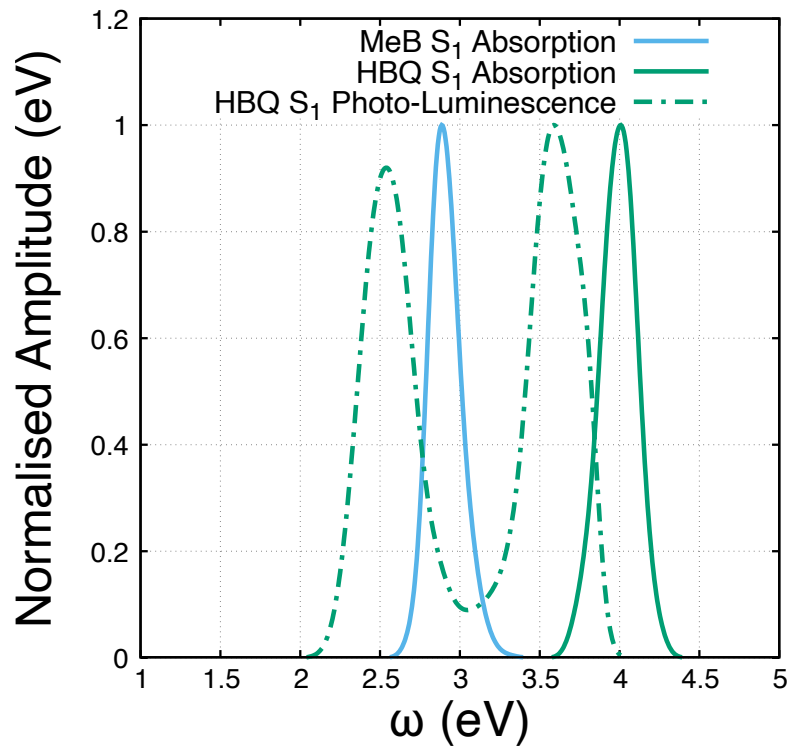

Figure S7: Normalized absorption and photoluminescence spectra of Methylene Blue and HBQ.

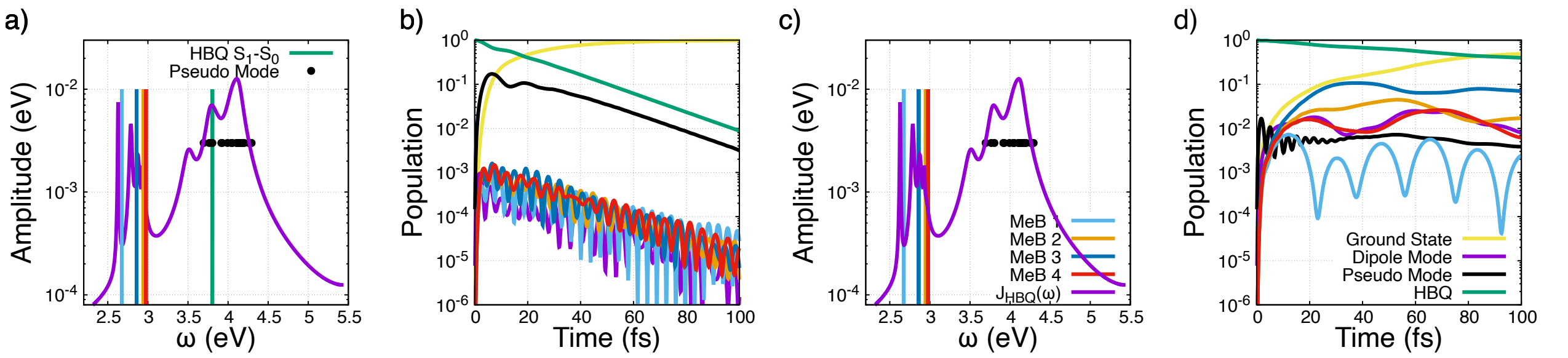

Figure S8: (a) Effective spectral density, perceived by HBQ, plotted together with the absorption spectra of Methylene Blue (light blue, orange, dark blue, and red). Black dots indicate the frequencies $\tilde{\omega}_k$ of the fitted modes that form the pseudo-mode in our model. (b) Time evolution of the excited-state population of the five emitters (HBQ green, MeB1 light blue, MeB2 orange, MeB3 dark blue, and MeB4 red) together with that of the dipolar mode (purple) and the pseudo-mode (black) of the nanoresonator. The ground-state population (yellow) increases steadily due to losses in the system. (c) and (d) same as panels (a) and (b), respectively, but after HBQ has undergone the intramolecular proton transfer reaction.

We performed simulations for investigating photochemistry-driven energy transfer within a two-level system treatment of the molecular emitters. As shown in Fig. S8b, such a treatment precludes energy transfer because of the lack of overlap in the initial excitation energies of both emitters (see spectrum in Fig. S8a). Running such TLS simulations with the central TLS corresponding to HBQ after the excited-state intramolecular proton transfer, i.e., central TLS resonant with the four peripheral ones, dismisses the large impact of the so-called pseudo-mode [8] in this setup (Fig. S8d) as we show in Fig. 5 of the main article.

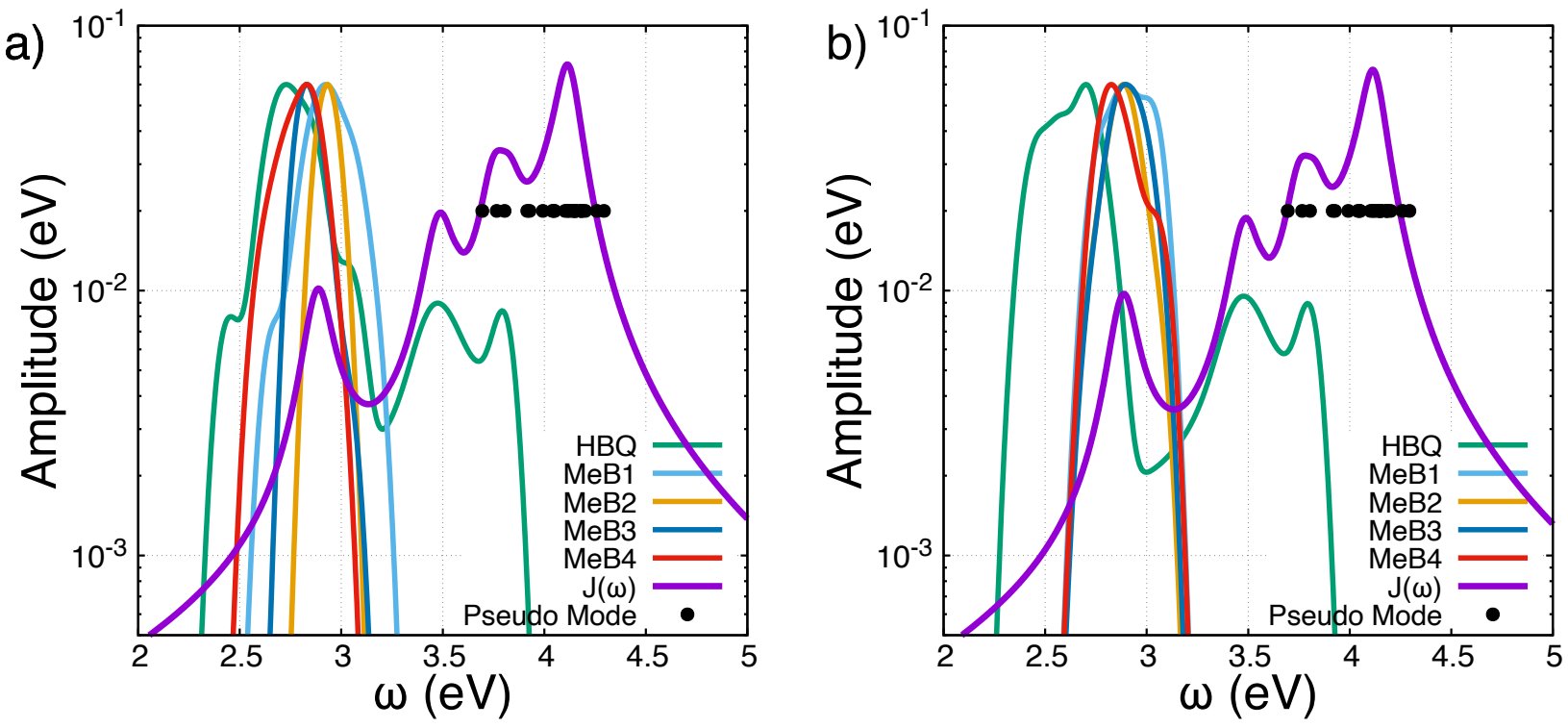

Figure S9: Spectral density of the silver nanoparticle dimer with five emitters in the center of its gap together with the normalized absorption and photoluminescence spectra of Methylene Blue and HBQ with (a) and without (b) initial disorder in the Methylene Blue ensemble.

For the MD simulation presented in the main article, dynamic disorder leads to a convenient overlap

between the dipole mode of the silver nanoparticle dimer, the absorption of Methylene Blue, and the emission of HBQ as depicted in Fig. S9a, in contrast to the same simulation but without initial diagonal/off-diagonal disorder in the Methylene Blue ensemble, Fig. S9b. Despite identical initial conditions for HBQ (same atomic positions and velocities in both simulations), HBQ Stokes shifts more significantly after the proton transfer reaction in the simulation without initial disorder in the Methylene Blue ensemble than in the simulation with initial disorder. This leads to reduced overlap of HBQ emission with the dipole mode of the nanoresonator and the absorption of Methylene Blue.

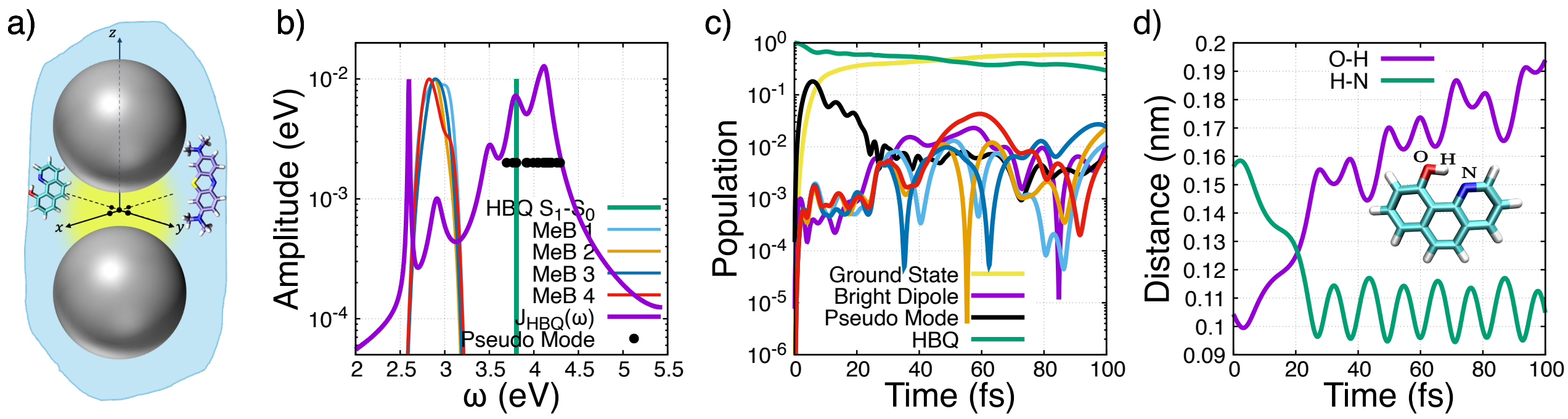

Figure S10: (a) Schematic representation of five molecular emitters within the gap of a silver nanoparticle dimer oriented along the z-axis. A molecular model of HBQ, depicted in the left inset, is positioned at the central spot while Methylene Blue molecules, depicted in the right inset, occupy the peripheral positions. (b) Effective spectral density, perceived by HBQ, plotted together with the absorption spectra of Methylene Blue (light blue, orange, dark blue, and red). Black dots indicate the frequencies $\tilde{\omega}_k$ of the fitted modes that form the pseudo-mode in our model. (c) Time evolution of the excited-state population of the five emitters (HBQ green, MeB1 light blue, MeB2 orange, MeB3 dark blue, and MeB4 red) together with that of the dipolar mode (purple) and the pseudo-mode (black) of the nanoresonator. The ground-state occupation (yellow) increases steadily due to losses in the system. (d) Distances between O, H, and N atoms of HBQ (inset). A distance of approximately 0.105 nm between H and N corresponds to a bonded state of the two atoms.

Indeed, when running a few-mode QM/MM MD simulation with idealized starting conditions where all Methylene Blue molecules have the same initial conformation and thus no initial diagonal/off-diagonal disorder (Fig. S10), the cavity-mediated intermolecular energy transfer between the initially excited HBQ and the four peripheral Methylene Blue molecules is not necessarily enhanced compared to a situation with initial disorder as shown in Fig. 5 of the main article. This highlights the paramount effect of dynamic disorder and consequently the need for dynamic approaches with which multiple trajectories can be run to sample the enormous phase space available to the system.

We conducted additional few-mode QM/MM MD simulations presented in Fig. S11. These additional results confirm the underlying mechanism involved in photochemistry-driven, cavity-mediated intermolecular energy transfer, with the efficiency depending on *(i)* the time at which the intramolecular proton transfer of HBQ completes and *(ii)* the overlap between the emission of HBQ, the resonant/off-resonant coupling to the dipolar mode of the nanoresonator, and the absorption of Methylene Blue. Importantly, this last factor is highly influenced by dynamic disorder.

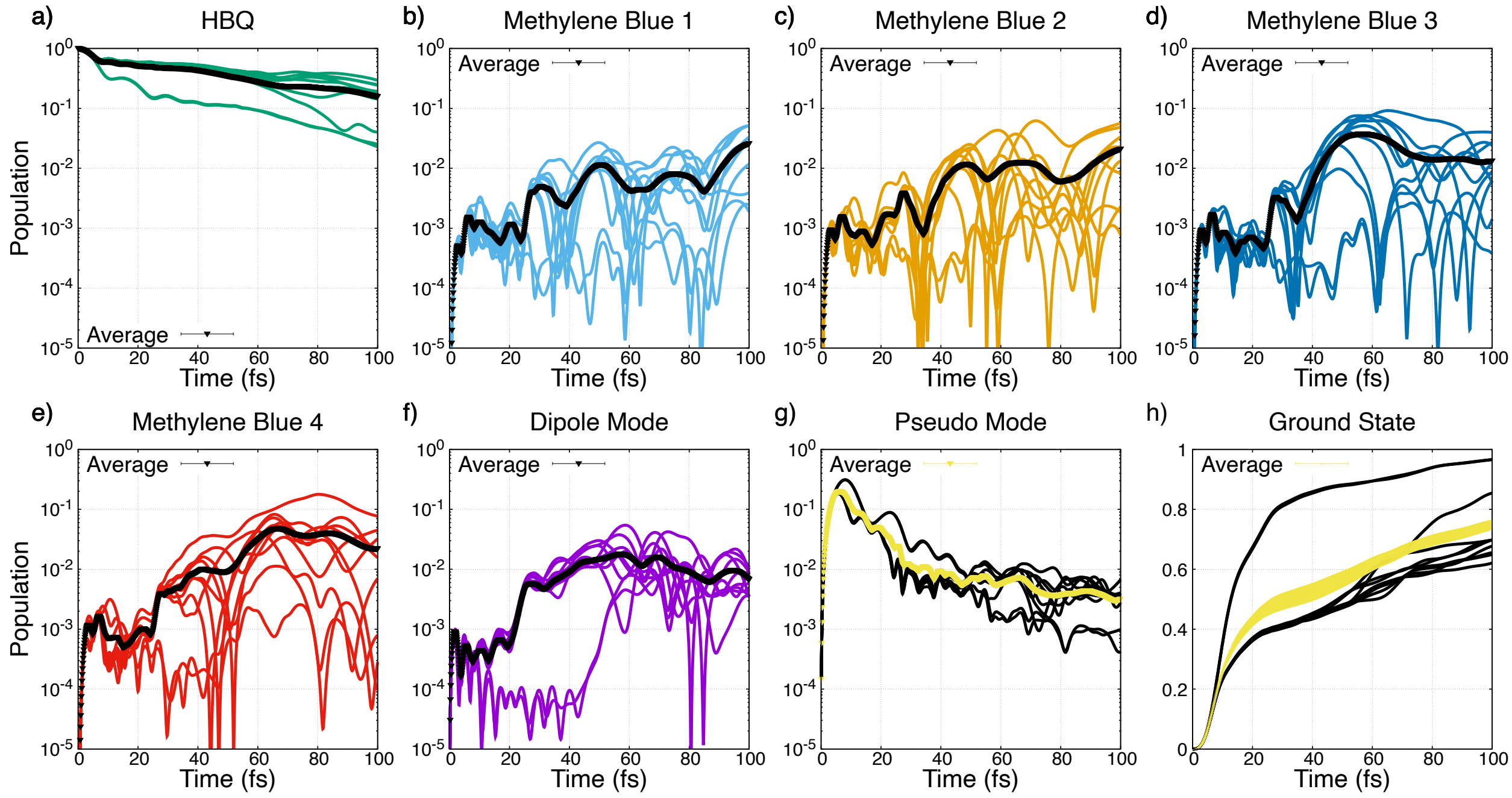

Figure S11: Time evolution of the excited-state population during 10 few-mode QM/MM MD simulations of (a) an HBQ molecule positioned at the central spot within the setup depicted in Fig. S10 and of (b), (c), (d), (e) Methylene Blue molecules at the peripheral positions. The average over these 10 realizations is plotted with black diamonds. Population dynamics of (f) the dipolar mode and (g) the modes constituting the broad pseudo-mode of the silver nanoparticle dimer. (h) Ground-state occupation due to losses of the silver nanoparticle dimer. Note the linear scale in this last plot. The average together with the standard deviation is plotted with yellow diamonds.

References

[1] D. Frenkel and B. Smit, *Understanding molecular simulation: from algorithms to applications*, Vol. 1 (Elsevier, 2001).

[2] A. Warshel and M. Levitt, "Theoretical studies of enzymatic reactions: dielectric, electrostatic and steric stabilization of carbonium ion in the reaction of lysozyme", J. Mol. Biol. **103**, 227–249 (1976).

[3] P. Ehrenfest, "Bemerkung über die angenäherte gültigkeit der klassischen mechanik innerhalb der quantenmechanik", Z. Phys. **45**, 445–457 (1927).

[4] I. Medina, F. García-Vidal, A. Fernández-Domínguez, and J. Feist, "Few-mode field quantization of arbitrary electromagnetic spectral densities", Phys. Rev. Lett. **126**, 093601 (2021).

[5] M. Sánchez-Barquilla, F. J. García-Vidal, A. I. Fernández-Domínguez, and J. Feist, "Few-mode field quantization for multiple emitters", Nanophotonics (2022).

[6] G. Granucci, M. Persico, and A. Toniolo, "Direct semiclassical simulation of photochemical processes with semiempirical wave functions", J. Chem. Phys. **114**, 10608–10615 (2001).

[7] H.-P. Breuer and F. Petruccione, *The Theory of Open Quantum Systems* (Oxford University Press, Oxford, 2007).

[8] A. Delga, J. Feist, J. Bravo-Abad, and F. J. Garcia-Vidal, "Quantum emitters near a metal nanoparticle: strong coupling and quenching", Phys. Rev. Lett. **112**, 253601 (2014).

[9] R. Tichauer, J. Feist, and G. Groenhof, "Multi-scale dynamics simulations of molecular polaritons : the effect of multiple cavity modes on polariton relaxation.", J. Chem. Phys. **154**, 104112 (2021).

[10] I. Sokolovskii and G. Groenhof, "Non-hermitian molecular dynamics simulations of exciton polaritons in lossy cavities", The Journal of Chemical Physics **160**, 092501 (2024).

[11] G. Bussi, D. Donadio, and M. Parrinello, "Canonical sampling through velocity rescaling", The Journal of Chemical Physics **126**, 014101 (2007).

[12]C. Gonzalez-Ballestero, J. Feist, E. Moreno, and F. Garcia-Vidal, "Harvesting excitons through plasmonic strong coupling", Phys. Rev. B **92**, 121402 (2015).
